# Immunoinformatics lessons on the current COVID-19 pandemic and future coronavirus zoonoses

**DOI:** 10.3389/fimmu.2023.1118267

**Published:** 2023-12-12

**Authors:** Daniel López, Marina García-Peydró

**Affiliations:** ^1^ Presentation and Immune Regulation Unit, Centro Nacional de Microbiología, Instituto de Salud Carlos III, Majadahonda, Spain; ^2^ Centro de Biología Molecular Severo Ochoa, Consejo Superior de Investigaciones Científicas (CSIC) - Universidad Autónoma de Madrid (UAM), Madrid, Spain

**Keywords:** HLA, vaccine, cross protection, SARS - CoV, immunoinformatic analysis

In 2021, “Oxfod-AstraZeneca COVID-19 Vaccine” and “Pfizer-BioNTech COVID-19 Vaccine” were the first vaccines to receive regulatory approval by European Medicines Agency (EMA) and Food and Drug Administration (FDA), respectively. However, despite this fast development, could another COVID-19 vaccine have been available more quickly? Since the early 20th century several SARS-CoV-1 vaccines, using inactivated SARS coronavirus or DNA constructions, were in the pipeline. These vaccines elicited strong immune responses and protective effects in preclinical models (1–3), and were well tolerated and produced both neutralizing antibodies and potent cellular immune responses in phase I clinical trials (4–6). Since the global eradication of the smallpox pandemic was possible through vaccination with other poxviruses of the same family, although the mutation rate of coronaviruses is much higher than that of poxviruses, would it be expected that vaccines against SARS-CoV-1 could protect against SARS-CoV-2, given the very close similarity between these two sarbecoviruses? (7).

The astonishing polymorphism of tens of thousands human leukocyte antigens (HLA) makes it extraordinarily difficult to experimentally test this hypothesis at the world population level. Nevertheless, these HLA alleles have been grouped into different HLA class I and II families, superfamilies, and supertypes that share strong similarities at the functional level of peptide-ligand specificity, relatively simplifying the problem. Thus, we used in silico approaches to overcome this challenge of herd immunity by analyzing the 600 most common HLA alleles covering > 90% of the world’s population regardless of ethnicity. We found that each of these most frequent HLA alleles could be able to present around 4-5 epitopes shared by SARS-CoV-1 and SARS-CoV-2 (8). Supporting our results, the scarce available data from slightly more than 200 SARS-CoV patients (limited to most prevalent and frequent HLA alleles) were highly concordant (91%) with our predicted epitopes (8). Because HLA genes are tightly linked in the genome, each individual expresses 6 to 12 different alleles (6 per chromosome) in a Mendelian fashion. Thus, a fully heterozygous person, as 85% of the world’s population is, could present up to 52 conserved viral epitopes (8), sufficient number to generate a complete cellular immune response. To support our argument, a recent study in mice demonstrated that immunization with SARS-CoV-1 vaccines induces, not only cytotoxic and helper T lymphocytes against SARS-CoV-2 as we hypothesized, but also some cross-reactive antibodies (9). Since most of the epitopes of neutralizing antibodies against the SARS-CoV-2 spike protein are not conserved in the SARS-CoV-1 sequence, it is likely that the cross-reactive adaptive immune response is due to T cells rather than antibodies, although serum from individuals vaccinated against SARS-Cov2 when transferred to mice provides relevant protection (9). Such partial cross-protective response of the complete adaptive immune system (9), also greatly reinforces our reasoning.

Based on the foreseeable cross-reactivity and consequent protection between sarbecovirus vaccines, those already existing against SARS-CoV-1 might have been included in phases 2 and 3 clinical trials at the beginning of the pandemic in 2020, and have contributed to reduce COVID-19 mortality since the end of 2020 until the approval of specific SARS-CoV-2 vaccines. The malaria vaccine is an interesting example of a WHO-approved vaccine that, although only partially protective (30% of severe disease), is expected to prevent tens of thousands of deaths per year (10). Therefore, the use of SARS-CoV-1 vaccines could have been very relevant in the fight against COVID-19.

For future pandemics, bioinformatics analyses could be a useful, fast and low-cost strategy to determine the potential cross-reactivity of currently available vaccines, compounds that could be used while specific vaccines against the new pathogen are being developed. And going even further, could this strategy be used preventively in the face of probable new pandemics related to current zoonoses? In addition to both sarbecoviruses, MERS-CoV, other betacoronavirus but included in the merbecovirus subgenus also caused zoonotic disease in 2017. Due to the evolutionary divergence between both subgenera, our bioinformatics analysis showed that there are no fully conserved epitopes between MERS-CoV and sarbecoviruses for any HLA allele (8). However, perhaps epitopes with some changes in their sequence could be recognized cross-reactively by a few T lymphocytes. This means that if a MERS-CoV-2 were to emerge, as has been the case with the SARS-CoVs, the current licensed vaccine against SARS-CoV-2 would probably not protect against this new merbecovirus.

Thus, a preventive MERS-CoV-specific vaccine could be developed by the WHO and other international organizations. This effort should include a Phase 1 clinical trial along with a comprehensive cellular immune response study for at least some of the most common HLA class I and class II alleles in the human population. All this could be done with by the with minimal expense in anticipation of a new merbecovirus zoonosis, because with a mortality rate of 35% like that of MERS-CoV, a very rapid development of a vaccine may be essential to avoid a dramatic “Don’t Look Up” scenario.

## Author contributions

All authors listed have made a substantial, direct, and intellectual contribution to the work and approved it for publication.
